# Competition among Supply Chains and Governmental Policy: Considering Consumers’ Low-Carbon Preference

**DOI:** 10.3390/ijerph15091985

**Published:** 2018-09-12

**Authors:** Xujin Pu, Zhiping Song, Guanghua Han

**Affiliations:** 1 School of Business, Jiangnan University, Wuxi 214122, China; puyiwei@ustc.edu (X.P.); szping66@hotmail.com (Z.S.); 2 School of International and Public Affairs, Shanghai Jiao Tong University, Shanghai 200030, China

**Keywords:** low-carbon production, consumers’ preference, cooperation, supply chain competition

## Abstract

Many manufacturers and retailers have cooperated for low-carbon production in various industries. This study examines the role of consumers’ low-carbon preference in this cooperation. We construct four scenarios to investigate the effects of consumers’ low-carbon preference on the market equilibrium of supply chains’ product selection strategy. Based on the game theoretic models, optimal solutions for the two supply chains are derived with different consumers’ preference for low-carbon products. Through the discussion, we uncovered the influence of consumers’ preference on price and demand and the relationship between the influence coefficient of retailers’ promotional effort on consumers’ utility and retailer profits. In addition, given the increase of government’s low-carbon production subsidy, two supply chains will both more likely choose low-carbon production. Interestingly, under the government subsidy, the profit of manufacturer will increase or decrease more than its retailer and the market structure will not change if the two supply chains have chosen low-carbon production.

## 1. Introduction

The 2017 report of the Intergovernmental Panel on Climate Change shows that the global emissions of greenhouse gases (GHG) have increased to unprecedented levels [1]. Consequently, the increasing rate of global temperature has increased significantly over the past few years. Thus, for sustainable and environmental production, activities must be taken by industries to reduce carbon emissions with the help of sustainable and environmental production (Barbier [2]; Nesticò and Sica [3]). Many countries are exerting their best effort to reduce carbon emissions. For example, Australia has promised to cut its carbon emissions by 5% by 2020 and 80 percent by 2050. The European Union has ensured that GHG emissions will be at least 20% lower in 2020 than they were in 1990. The Chinese government has also pledged to reduce emissions per unit of GDP by 60 to 65% from 2005 levels by 2030. Moreover, a substantial increase in consumers’ environmental awareness facilitates the protection of the environment. An increasing number of consumers are concerned with products’ environment performance and show low-carbon preference to green consumption. Accordingly, products with low carbon emissions would have a considerably high market share, reputation, and market value. These factors prompt enterprises in all industrial sectors to reduce emissions. A total of 30 leading companies, including Volvo, Coca-Cola, and Yingli Solar, have participated in the WWF Climate Savers Program and promised a large-scale reduction of carbon emissions [4].

Given that the market competition has become increasingly fierce, the cooperation between the supply chain’s upstream (manufacturer) and downstream (retailer) is also becoming considerably important to the supply chain. The competition among manufacturers is changing to the competition between supply chains (Christopher [5]; Ai et al. [6]). For example, Ford and GM, together with their respective retailers, sell cars to consumers to obtain maximum profit. Apple and Xiaomi sell mobile phones through their respective retailers and generally compete with other mobile phone companies. Manufacturers and retailers may also collaborate to use “low carbon” as their selling point. For example, Gome cooperates strategically with Haier to encourage the latter to implement low-carbon production and actively guide consumers on low-carbon consumption to reduce carbon emission. By cooperating with each other, Haier and Gome’s profits have been increasing despite the general downturn in demand. Given the consumers’ low-carbon preference and the competition between the two supply chains, the future of the market equilibrium is an interesting point of research.

However, low-carbon production can be costly because it needs additional resources, including specialized equipment, additional inputs, and complicated human resource management. If the premium that consumers are willing to pay is insufficiently large to cover the additional costs, then subsidy, which is the most common instrument for governments, is the key to promote low-carbon products. Many countries, including the US, Japan, Germany, and France, have subsidized car companies to produce new energy vehicles. Effective policies can regulate and guide the behaviors of supply chains in the market and also promote social sustainable development. However, if the subsidy is inappropriate, then such instrument will damage the market structure. Thus, it is important for the government to know what the market equilibrium between the two supply chains will be with the increment of government subsidy. This study will also focus on this topic.

In this study, chain-to-chain competition is the competition between two supply chains, with each chain consisting of a manufacturer and its exclusive retailer. We use game theoretic models to analyze the chain-to-chain competition and obtain market equilibrium of the two supply chains. Nash equilibrium, one of most essential ingredients in game theory named after American mathematician John Forbes Nash Jr., is a solution concept of a non-cooperative game involving two or more players. In Nash equilibrium, each player is assumed to know the equilibrium strategies of the other players, and no player has anything to gain by changing their own strategies. In this study, we consider the competition between two different supply chains, the decision conditions of each participators are public information and known to all supply chain members. Therefore, the supply chains we considered in this paper compete following a Nash equilibrium game process. In particular, we employ Nash equilibrium to formulate the decision process and provide insights into the following questions: (1) What are the effects of consumers’ low-carbon preference on the market equilibrium of supply chains’ product selection strategy? (2) What is the influence of consumers’ preference on price and demand? (3) What is the relationship between the influence coefficient of retailers’ promotional effort on consumers’ utility and retailer profits? (4) What is the impact of government subsidy on the market structure of two supply chains? 

The supply chains and government can benefit from this study. On the one hand, the supply chains can obtain an improved understanding of the effects of consumers’ low-carbon preference and achieve a good market equilibrium. On the other hand, the government can learn the influences of low-carbon production subsidy on supply chains’ product selection. The remainder of this paper is organized as follows. Section 2 positions the relevant literature on low-carbon production and chain-to-chain supply competition. Section 3 provides the model formulation and assumptions. Section 4 presents four models on the manufacturers’ production selection and discusses the results. Section 5 extends to investigate the effects of government subsidy on low-carbon production. Section 6 summarizes our main findings and discuss opportunities for further research.

## 2. Literature Review

Our study is related to two research streams that are briefly reviewed as follows. The first stream is the literature review on manufacturers’ low-carbon production. Among the early studies, Zhao et al. [7] investigated the impact of allowance allocation systems in markets and obtained an equilibrium production based on cap-and-trade setting under a perfect competition. Benjaafar et al. [8] studied the impact of operational decisions on carbon emissions by incorporating carbon emission considerations (e.g., carbon caps, carbon taxes, and carbon offsets) into simplified inventory models, as well as constructed mixed-integer linear programming models to determine the associated costs, thereby providing a potential template for further study. Du et al. [9] incorporated consumer preference into low-carbon production management and analyzed the effects of carbon emissions on a supply chain. Tang et al. [10] analyzed three approaches related to the effects of controlling carbon emissions in transportation and inventory management. Drake et al. [11] studied the impact of emission tax and emission cap-and-trade regulation on a firm’s technology choice and capacity decisions. Fan and Dong [12] investigated how the government can select a subsidy strategy in low-carbon diffusion by considering heterogeneous agents’ behavior. Du et al. [13] proposed a carbon-related price–discount sharing-like scheme to achieve channel coordination. Meng et al. [14] investigates product selection strategies of two competitive firms in the presence of carbon tax and conducts an analytical examination of the effect of power structure on the firm’s product selection strategy over different levels of carbon tax rate.

Another relevant stream of literature explores the competition among supply chains. McGuire and Staelin [15] contributed immensely in this area by investigating the equilibrium supply chain structures in a duopoly market, in which two manufacturers compete with each other by selling products through their exclusive retailers. McGuire and Staelin showed that for the majority of specifications, product substitutability does influence the equilibrium distribution structure. Thereafter, McGuire and Staelin’s research has been extended by other researchers. For example, Xiao and Choi [16] studied how the channel structure strategies and wholesale prices of manufacturers depend on the risk sensitivity, pricing power, and purchasing option of retailers. Xiao and Yang [17] developed a price-service competition model of two supply chains to investigate the optimal decisions of players under demand uncertainty; they determined that the higher the risk sensitivity of one retailer, the lower his optimal service level and retail price will be. They also determined that the effects of a rival’s risk sensitivity on the retailer’s decisions depend on the substitutability of the two products. Zhao and Shi [18] incorporated contracting strategy into the supply chain structure selection problem. Accordingly, they determined that decentralized supply chains perform better under strong market competition, whereas integrated supply chains perform better when many suppliers exist. Mahmoodi and Eshghi [19] introduced demand uncertainty to investigate which supply chain structure is preferable in an industry consisting of two distinct supply chains that compete with each other over price. Amin-Naseri and Khojasteh [20] developed a price competition model under a demand uncertainty environment between two leader-follower supply chains. Each supply chain consists of one risk-neutral manufacturer and one risk-averse retailer. Thereafter, the optimal wholesale and retail prices for the leader and follower supply chains are obtained under various supply chain network structures. Taleizadeh et al. [21] considered two competing supply chains, in which both chains launch the same product (under different brands) to the market by applying different composite coordinating strategies. The researchers aimed to determine the optimal selling prices and the order quantities of the manufacturer and retailers in each chain in the presence of different composite coordinating strategies. Wang et al. [22] incorporated markup pricing strategies into the chain-to-chain supply competition and determined that the equilibrium pricing strategy depends on the level of chain-to-chain supply competition.

Evidently, the management of low-carbon supply chains is becoming an important research topic in recent years. However, the related studies either only analyze consumers’ low-carbon preference on manufacturers or merely investigate the completion between supply chains. To the best of our knowledge, no previous supply chain study has incorporated consumers’ low-carbon preference into the competition among supply chains. Therefore, this topic is of interest for further research. In addition, the effect of the government’s low-carbon production subsidy on the competitive market structure is included in our research.

## 3. Model Description

**Figure 1:**
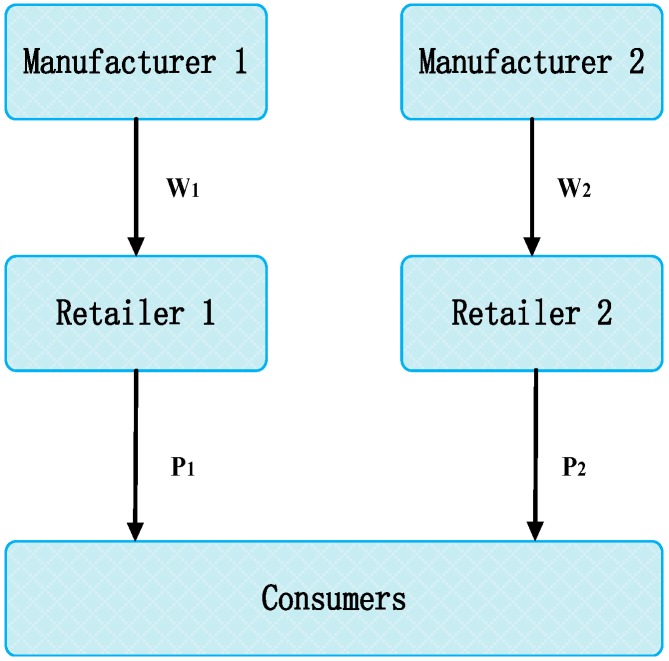
Structure of the manufacturing supply chain under duopoly model.

This study considers two competitive supply chains (i.e., supply chains 1 and 2) in a duopolistic market. Supply chain 1 consists of manufacturer 1 and retailer 1, while supply chain 2 comprises manufacturer 2 and retailer 2. The manufacturers are the leaders in the supply chain and decide whether to produce low-carbon or regular products. If the manufacturer chooses to produce low-carbon products, then its own retailer will choose to promote them. The low-carbon and regular products are completely substitutable in a competitive market. For example, IKEA and the World Wildlife Fund (WWF) work together to reduce greenhouse gas (carbon dioxide) emissions from IKEA’s production operations. The project includes improving energy efficiency and enabling IKEA suppliers to use renewable energy. Apple claims that all its facilities around the world, including Apple’s offices, retail stores and data centers, are now powered entirely by clean energy. In order to reduce greenhouse gas emissions and act against climate change more efficiently, Apple also requires its partners to use clean energy. The low-carbon products of IKEA and Apple can be replaced by their competitors who choose regular products. In this paper, we consider the same products (e.g., IKEA product) are made by two different manufacturers, both of which choose low-carbon production or regular-carbon production. Both the products made by low-carbon production and regular-carbon production are available to markets and consumers’ consumption is directly affected by their low-carbon preference. Figure 1 illustrates the schematics for the problem.

Consumers’ valuation for a regular product is *V* (*V* > 0), where *V* is sufficiently large. The market size of the product is 1 and consumers are uniformly distributed over [0, 1] with two retailers at opposite ends. When consumers come to any one retailer to buy products, the travelling cost is *t* (*t* > 0) per unit distance. Consumers prefer low-carbon products because of their awareness for protecting the environment. The consumers’ preference for low-carbon products is assumed as *τ* (*τ* > 0). We use *e* (0 < *e* < 1) to denote the carbon emission reduction rate of low-carbon products, while the carbon emission reduction rate of regular products is 0. The premium that consumers are willing to pay for low-carbon products is *τe*.

The cost of regular production is *c_i_*, *i* = 1, 2. If the manufacturer wants to produce low-carbon products, then he/she must invest money to employ low-carbon technologies. The quadratic function form is extensively adopted to describe the cost pattern in the literature (e.g., Yao and Liu [23]; Atasu et al. [24]). Accordingly, the cost of carbon emission reduction will accelerate to achieve a high level of emission reduction and the additional cost is kie22, *i* = 1, 2, where *k_i_* represents the per unit cost to adopt low-carbon production. Assume that the per unit cost to adopt low-carbon production is lower for manufacturer 1 than for manufacturer 2 (*k*_1_ > *k*_2_). Because the retailer’s low-carbon promotion incurs extra a few additional costs, we also use quadratic function to describe it. That is, c(θ)=δθi22, *i* = 1, 2, where *θ_i_* denotes the retailer’s promotional effort when the manufacturer chooses low-carbon production. Without loss of generality, we assume that *θ*_1_ > *θ*_2_. *δ* (*δ* > 0) is the influence coefficient of the retailers’ promotional effort on consumers’ utility. Table 1 summarizes these notions.

Each supply chain has two choices, namely, *N* (regular) and *L* (low-carbon), for their product strategies. Thus, a total of four scenarios are used for their product selections, which are denoted as follows:

*Scenario NN*. Two manufacturers choose to produce regular products. The net utilities that a consumer derives from buying from two retailers are $U_{1}^{NN}=V-p_{1}^{NN}-tx$ and $U_{2}^{NN}=V-p_{2}^{NN}-t\left(1-x\right)$.

*Scenario NL*. Manufacturers 1 and 2 choose to produce regular and low-carbon products, respectively. The net utilities that a consumer derives from buying regular and low-carbon products are $U_{1}^{NL}=V-p_{1}^{NL}-tx$ and $U_{2}^{NL}=V-p_{2}^{NL}-t\left(1-x\right)+\tau e+\xi \theta_{2}$, respectively.

*Scenario LN*. Manufacturers 1 and 2 choose to produce low-carbon and regular products, respectively. The net utilities that a consumer derives from buying low-carbon and regular products are $U_{1}^{LN}=V-p_{1}^{LN}-tx+\tau e+\xi \theta_{1}$ and $U_{2}^{LN}=V-p_{2}^{LN}-t\left(1-x\right)$, respectively.

*Scenario LL*. Two manufacturers choose to produce low-carbon products. The net utilities that a consumer derives from buying products from two retailers are $U_{1}^{LL}=V-p_{1}^{LL}-tx+\tau e+\xi \theta_{1}$ and $U_{2}^{LL}=V-p_{2}^{LL}-t\left(1-x\right)+\tau e+\xi \theta_{2}$.

Table 2 presents the game matrix between supply chains 1 and 2.

## 4. Equilibrium Solutions and Discussions

As stated previously, we are interested in the influence of consumers’ low-carbon preference on supply chains’ low-carbon production strategy, wholesale price, retail price as well as market demand. Meanwhile, retailer’s promotion behavior and governments’ influence on the market structure might affect consumers’ utility and retailer’s profits, we analytically explore their relations in this section. Since the supply chain partners act following a N ash pricing games, we answer the above questions with Nash game processes and present the mathematical results by Table A1, Table A2, Table A3 and Table A4 in Appendix A. Based on the mathematical results, we employ four lemmas and three propositions to illustrate our findings regarding our concerns. We first present the following proposition to characterize the manufacturer’s optimal response strategies within two supply chains.

**Proposition** **1.** *For the product selection strategies of the two manufacturers, a strategy equilibrium NN exists if τ < A, a strategy equilibrium LL exists if τ ≥ B, and a strategy equilibrium LN exists if A ≤ τ < B, we denote* $A=\frac{1}{2}k_{1}e-\frac{\xi \theta_{1}}{e}$ *and* $\;B=\frac{1}{2}k_{2}e-\frac{\xi \theta_{2}}{e}$ *.*

**Proof.**  See Appendix B.

**Figure 2:**
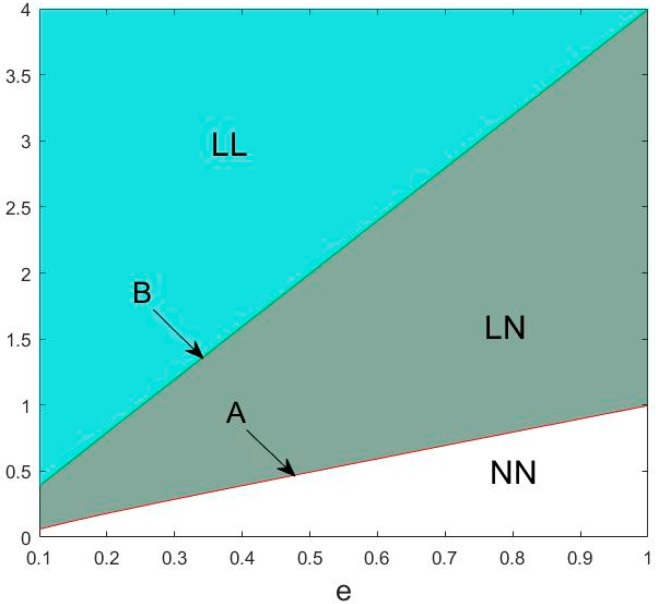
Division of the market structure.

Proposition 1 shows that when the low-carbon environmental awareness that consumers have is not evident, both manufacturers in the market will choose to produce regular products (Figure 2). When consumers’ low-carbon environmental awareness increases, the manufacturer with low cost of low-carbon production will choose low-carbon production, whereas the other manufacturers will choose regular production. When consumers actively pursue low-carbon products, both manufacturers will choose to produce low-carbon products. Consequently, environmental awareness is the key factor. The government should encourage low-carbon product consumption as a way to improve low-carbon production with market demand. This method is crucial to urge manufacturers to produce low-carbon products.

**Figure 3:**
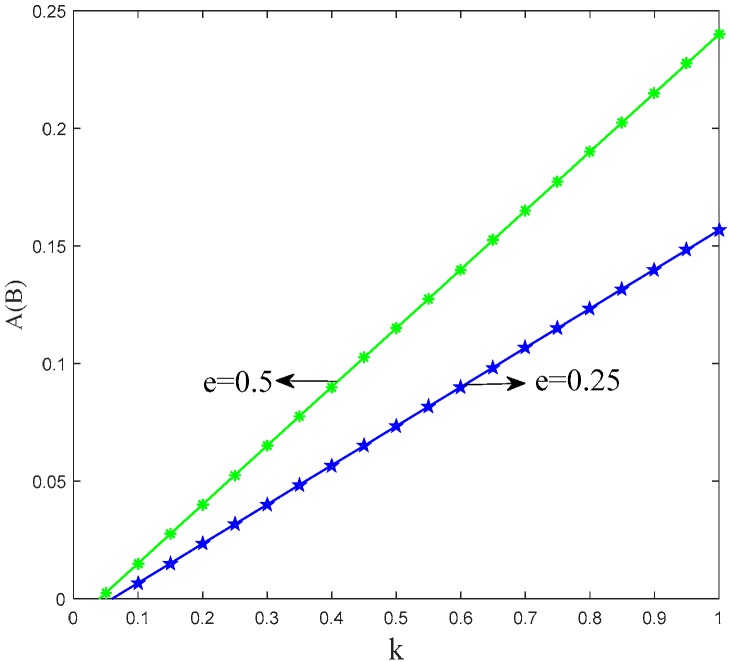
Trend of *A*(*B*) with *k*.

**Figure 4:**
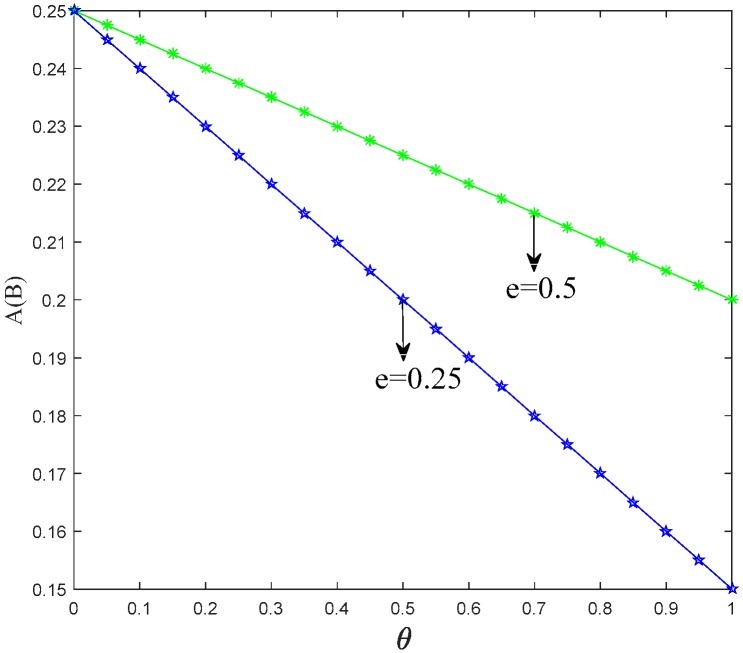
Trend of *A*(*B*) with *θ*.

**Figure 5:**
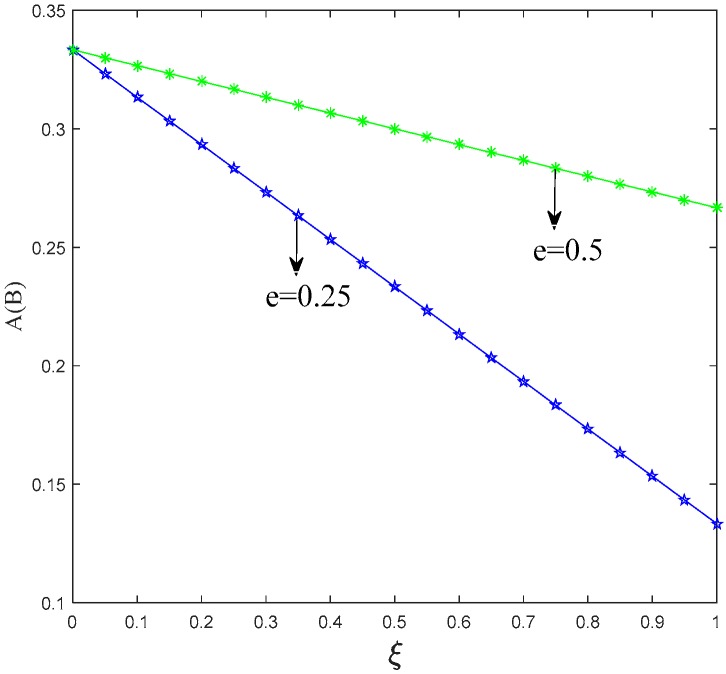
Trend of *A*(*B*) with *ξ*.

After investigating the trend of *A*(*B*) in Proposition 1, the threshold of two manufacturers choosing different production strategies is positively associated with the unit cost of low-carbon production. The threshold also increases with the increment of the carbon emission reduction rate. Meanwhile, the threshold has negative correlation with the retailers’ low-carbon promotional efforts and the effect of low-carbon promotion on the level of consumer utility. Figure 3, Figure 4 and Figure 5 show the trend of *A*(*B*) in relation to the other factors.

Figure 3, Figure 4 and Figure 5 illustrate that it is the result of superposition of multiple factors for manufacturers to choose production strategy. Manufacturers can produce more low-carbon products via reducing their low-carbon production cost and strengthening the low-carbon promotion effort of their retailers.

The demand gap of the two supply chains will change with the increment of consumers’ preference coefficient. We can derive Lemma 1 after conducting an investigation.

**Lemma** **1.** *Let $\Delta D^{NN}=D_{1}^{NN}-D_{2}^{NN},\;\Delta D^{LN}=D_{1}^{LN}-D_{2}^{LN}$, and $\Delta D^{LL}=D_{1}^{LL}-D_{2}^{LL}$. If τ > A, then $\Delta D^{NN}<\Delta D^{LN}<\Delta D^{LL}$; if A ≤ τ < B, then $\Delta D^{NN}\leq \Delta D^{LN}<\Delta D^{LL}$; and if τ ≥ B, then $\Delta D^{NN}\leq \Delta D^{LN}\leq \Delta D^{LL}$.*

**Proof.**  See Appendix B.

**Figure 6:**
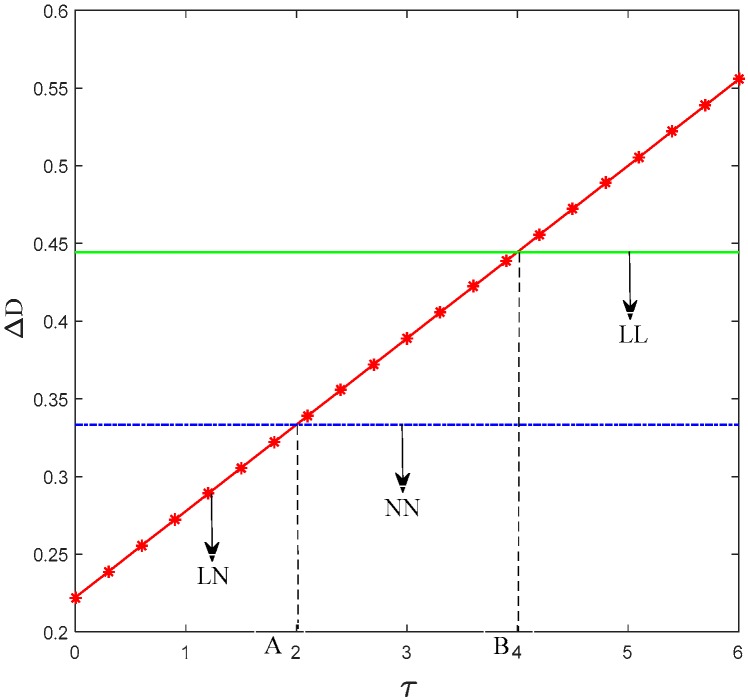
Trend of Δ*D* with *τ*.

Figure 6 shows the relationship between the consumers’ preference coefficient and the demand gap of the two supply chains. Moreover, Figure 6 shows that when the consumers’ low-carbon preference is relatively weak, if two manufacturers both produce low-carbon products, then the shortfall between their market share is the biggest. If manufacturers 1 and 2 choose to produce low-carbon and regular products, respectively, then the difference between their market share is minimal.

This phenomenon illustrates that if consumers’ low-carbon preference is relatively weak, then manufacturer 1 has a small competitive advantage. In this situation, when both manufacturers choose to produce low-carbon products, no significant difference is observed between the two types of products. Then the competition between two supply chains is transformed into the promotion competition and the manufacturer will have the advantage when its retailer has larger low-carbon promotional efforts. If the influence of consumers’ low-carbon preference on their purchasing behavior is big, the manufacturer who chooses low-carbon production will have a huge market competitive advantage.

**Proposition** **2.** *If τ < B, then* $p_{1}^{NN*}<p_{1}^{LN*}<p_{1}^{LL*}$ *; if τ ≥ B, then* $p_{1}^{NN*}<p_{1}^{LL*}<p_{1}^{LN*}$ *.*

**Proof.**  See Appendix B.

**Figure 7:**
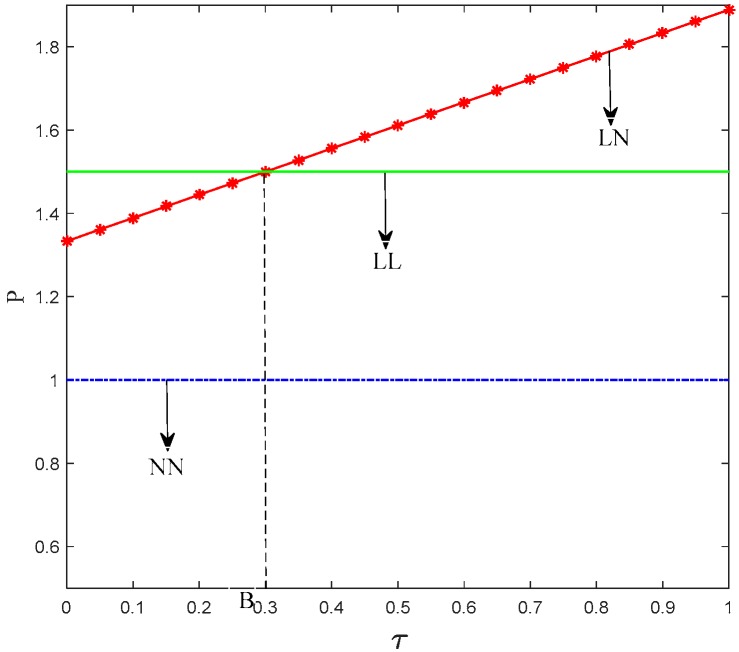
Trend of *p_i_* with *τ*.

Proposition 2 indicates that when products’ carbon emission reduction has low impact on the consumer purchase decision, the price of low-carbon product from manufacturer 1 is the largest in the LL scenario. When the impact of products’ carbon emission reduction on the consumer purchase decision exceeds a certain level, low-carbon products from manufacturer 1 have the largest price in the LN scenario. This finding implies that consumers’ low-carbon preference can affect the price that they are willing to pay. If low-carbon products on the market is all from manufacturer 1 and consumers’ preference for low-carbon products is large, then the retailer 1 will increase the prices. Moreover, the price of low-carbon products is always higher than that of regular products regardless of consumers’ preference because the former costs more. Figure 7 shows the trend of the price of retailer 1 to the consumers’ preference coefficient.

Thereafter, we investigate the demand of manufacturer 1 in three scenarios.

**Lemma** **2.** *When τ < A, then*$D_{1}^{LN}<D_{1}^{NN}<D_{1}^{LL}$*; when A ≤ τ < B,*$D_{1}^{NN}<D_{1}^{LN}<D_{1}^{LL}$, *when τ ≥ B,*$D_{1}^{NN}<D_{1}^{LL}<D_{1}^{LN}$*.*

**Proof.**  See Appendix B.

**Figure 8:**
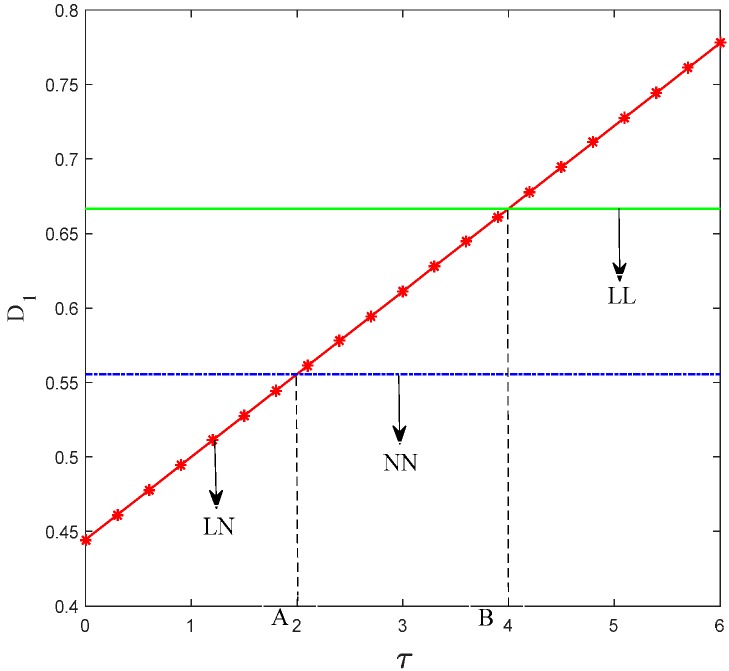
Trend of *D*_1_ with *τ*.

Lemma 2 indicates that if consumers’ preference on low-carbon products is relatively weak and only manufacturer 1 chooses to produce low-carbon products, then the market share between the two supply chains is the smallest. When consumers’ preference on low-carbon products increases, manufacturer 1 gradually achieves a large market share with the expansion of its competitive advantage. Figure 8 shows the trend of manufacturer 1’s demand to consumers’ preference coefficient.

Evidently, the market share of manufacturer 1 is larger in scenario LL than in scenario NN regardless of the low-carbon preferences of consumers. The reason is that in scenario LL, manufacturer 1 has a lower low-carbon production cost than that of manufacturer 2 and retailer 1 also devotes more promotion effort than retailer 2.

**Lemma** **3.** $\frac{\Pi_{R1}^{LN}}{\delta \xi }>0,\;\frac{\Pi_{R2}^{LN}}{\delta \xi }<0,\;\frac{\Pi_{R1}^{LL}}{\delta \xi }>0,\;\frac{\Pi_{R2}^{LL}}{\delta \xi }<0.$

**Proof.**  See Appendix B.

**Figure 9:**
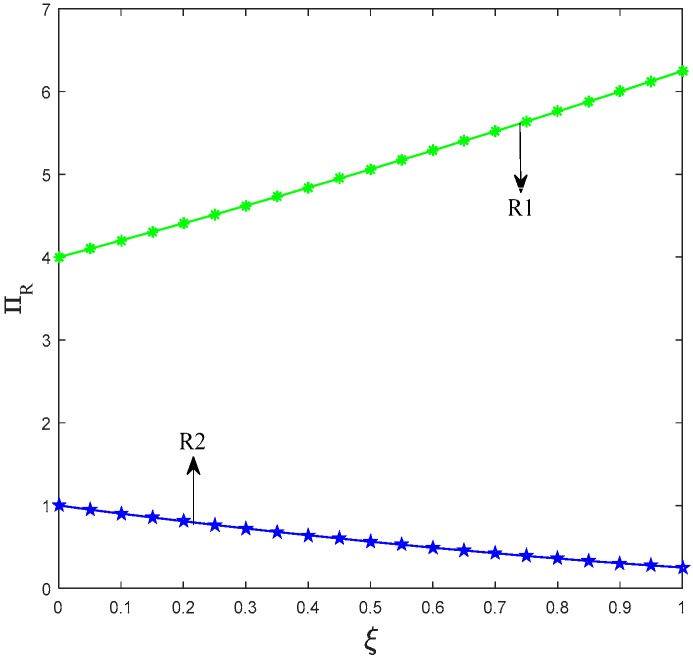
Trend of $\Pi_{R}^{LN}$ with *ξ*.

**Figure 10:**
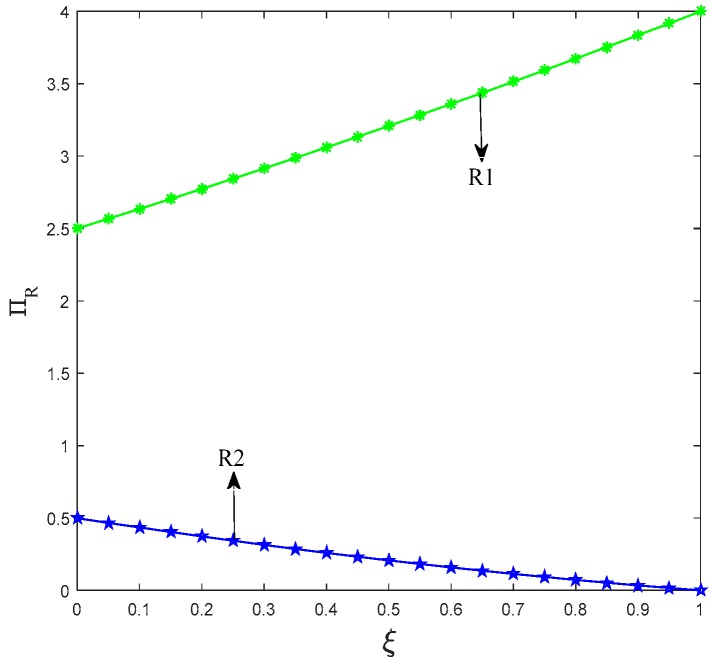
Trend of $\Pi_{R}^{LL}$ with *ξ*.

Lemma 3 presents that in scenarios LN and LL, the profit of retailer 1 is positively related to its influence of promotional effort on consumer utility, whereas the profit of retailer 2 is negatively related to it. That is, when only one manufacturer chooses to produce low-carbon products, if the influence of promotional efforts on consumers is big, then consumers have more tendency to buy low-carbon products and the retailer will receive more profits. In the situation where both manufacturers choose to produce low-carbon products, the promotion effort of retailer 1 is larger than that of retailer 2. Therefore, when the influence of promotional effort of low-carbon production on consumers is large, the retailer 1 receives more profits. Figure 9 and Figure 10 show the trend of the profit of retailers to the influence coefficient of the promotional effort on consumers’ utility.

**Lemma** **4.** $\frac{\delta p_{1}^{LL}}{\delta e}>0,\;\frac{\delta p_{2}^{LL}}{\delta e}>0,\;\frac{\delta p_{1}^{LN}}{\delta e}>0.\;$

**Proof.**  See Appendix B.

Lemma 4 indicates that the price of a low-carbon product is positively correlated with its carbon emission rate. The reason is that low-carbon production means that companies need to pay additional costs, such as using clean energy and low-carbon materials. When the carbon emission rate of low-carbon products is high, the cost will be high and the price of low-carbon products will also be high. The government should set suitable carbon emission standards for low-carbon products based on the actual conditions of enterprises.

## 5. Governmental Subsidy Policy

The model analyzed in Section 4 and Section 5 only discusses the low-carbon production selection strategy of two supply chains. However, in reality, governments constantly use subsidy policies to promote low-carbon production for controlling carbon emissions. Accordingly, the government is assumed to provide a subsidy at a rate of *λ* (*λ* > 0) for each unit of carbon emission. Compared with the original model in Section 4, in scenario NL, the other remains unchanged, while the profit of manufacturer 2 changes to $\Pi_{M2}^{NL}=\left(w_{2}^{NL}-c_{2}-\frac{1}{2}k_{2}e^{2}+\lambda e\right)D_{2}^{NL}$. In scenario LN, the other remains unchanged, while the profit of manufacturer 1 changes to $\Pi_{M1}^{LN}=\left(w_{1}^{LN}-c_{1}-\frac{1}{2}k_{1}e^{2}+\lambda e\right)D_{1}^{LN}$. In scenario LL, the profits of manufacturers 1 and 2 change. That is, $\Pi_{M1}^{LL}=\left(w_{1}^{LL}-c_{1}-\frac{1}{2}k_{1}e^{2}+\lambda e\right)D_{1}^{LL}$ and $\Pi_{M2}^{LL}=\left(w_{2}^{LL}-c_{2}-\frac{1}{2}k_{2}e^{2}+\lambda e\right)D_{2}^{LL}$. In scenario NN, the equilibrium will not be affected by a government subsidy. We summarize all these results in Table 3, Table 4 and Table 5.

For simplicity, let $\bar{A}=\frac{1}{2}k_{1}e-\frac{\xi \theta_{1}}{e}-\lambda$ and $\bar{B}=\frac{1}{2}k_{2}e-\frac{\xi \theta_{2}}{e}-\lambda$. The following proposition characterizes the optimal response strategies of two supply chains’ leader (manufacturer) with government subsidy policy.

**Proposition** **3.** *Given government’s low-carbon production subsidy, the dividing lines of two manufacturers’ optimal response strategies changes from* A *and* B *into* $\bar{A}$ *and* $\bar{B}$ *. Moreover,* $A\leq \bar{A},\;B\leq \bar{B}$ *.*

**Figure 11:**
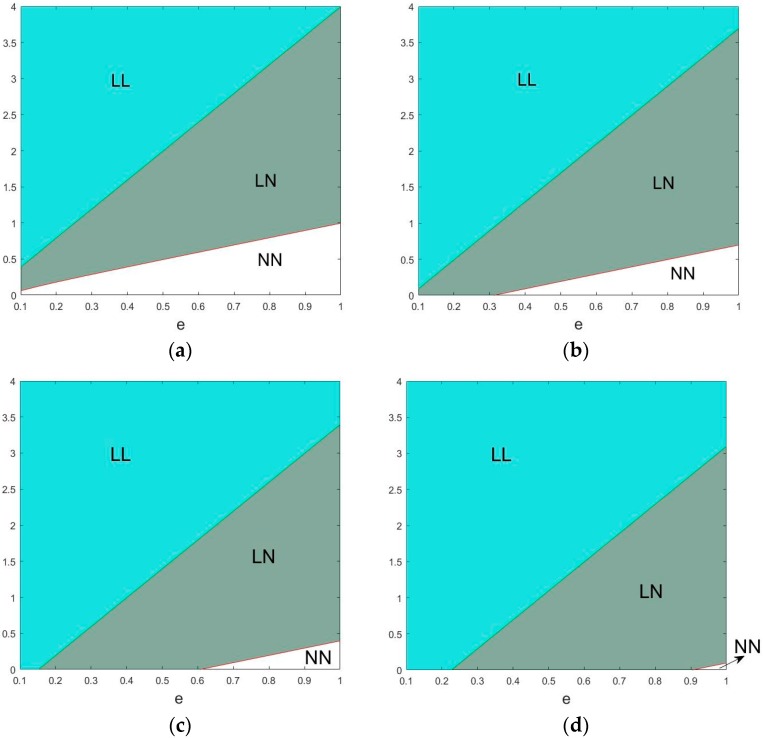
Changes of the market structure (**a**) *λ* = 0 (**b**) *λ* = 0.3 (**c**) *λ* = 0.6 (**d**) *λ* = 0.9.

Proposition 3 illustrates that given government’s low-carbon production subsidy, two manufacturers are more likely to choose scenarios LL and LN and less likely to choose scenario NN. This proves that subsidy is a beneficial tool for governments to regulate and guide the behavior of supply chains in the market. To reduce carbon emissions, governments should subsidize the low-carbon products. Figure 11 shows the changes of the market structure with certain value of subsidy.

Thereafter, we investigate the specific effect of subsidy on the supply chain. We find that when only one manufacturer chooses low-carbon production (scenarios NL and LN) and the government implements low-carbon production subsidy policy:

(1) The low-carbon supply chain’s wholesale price and sale price will decrease and the decrement is more than the wholesale price and sale price of regular supply chain.
(2) The profit of the manufacturer who chooses low-carbon production and its retailer will both increase, whereas the profit of the manufacturer that chooses regular production and its retailer will both decrease.
(3) The profit of a manufacturer will increase or decrease more than that of its retailer.

The above conclusions imply that when only one low-carbon supply chain exists in the market, the government’s low-carbon subsidy has a huge effect on both supply chains. The subsidy will cause the low-carbon manufacturer to decrease its wholesale price to its retailer, thereby leading to the decrement of retailer’s sale price to the consumers. This situation will also force the other supply chain to decrease its wholesale price and sale price. However, the decrement is smaller than that of low-carbon supply chain. Although the decrement of wholesale price and sale price, the low-carbon manufacturer and its retailer receive more profits with the government’s subsidy, whereas the regular manufacturer and its retailer receive less profit and are forced to loss some market share. As the leader of the supply chain, the manufacturer takes more responsibility and the change in profit of manufacturer is bigger than its retailer during the competition.

We also find that when two manufacturers choose low-carbon production (scenario LL), the government’s low-carbon production subsidy has no influence on the market structure. Moreover, the government’s low-carbon production subsidy will be totally acquired by consumers and the profit of the manufacturers and retailers remain the same as the situation without subsidy. This conclusion illustrates that if the two supply chains choose low-carbon production, then the government’s subsidy loses its effect on the market structure and is only beneficial for consumers. Therefore, government subsidy is not suggested when two supply chains have chosen low-carbon production.

## 6. Conclusions and Future Directions

This study incorporates consumers’ low-carbon preference into the supply chain competition. Analytical models are constructed to investigate the Nash pricing game. The main results are as follows:

(1) When consumers’ low-carbon preference is low, two manufacturers will choose regular production. When consumers’ low-carbon preference increases, the manufacturer with low cost of low-carbon production will choose low-carbon production, whereas the other manufacturer will still choose regular production. When consumers have a high preference for low-carbon products, both manufacturers will choose low-carbon production.
(2) When products’ carbon emission reduction has a low impact on the consumer purchase decision, the price of low-carbon product from manufacturer 1 is the largest in scenario LL, only manufacturer 1 choose low-carbon production, and the market share between the two manufacturers is the smallest. When the impact of products’ carbon emission reduction on consumers exceeds a certain level, low-carbon products from manufacturer 1 have the largest price in scenario LN and manufacturer 1 gradually achieves a large market share with the expansion of its competitive advantage.
(3) If the manufacturer with low fare to reduce carbon emissions chooses to take low-carbon production, regardless of what the other manufacturer chooses, then the profit of its retailer is positively linked to the influence coefficient of promotional efforts on consumer utility, whereas the other retailer’s profit is negatively linked to it.
(4) Given the increase of government’s low-carbon production subsidy, two manufacturers will more likely choose scenarios LL and LN and less likely choose scenario NN. This proves that government subsidy have an important role to play in reducing carbon emissions.

However, a few limitations are presented in this study. First, we assume that consumers’ low-carbon preference is the same for every consumer. An interesting extension is to consider the case where consumers have different low-carbon preference and thus we can further discuss what effect it will have when consumers’ low-carbon preference is represented by quadratic function. Second, this study assumes the demand function to be deterministic. It will be interesting to conduct research when consumers’ demand is stochastic. Third, the current research supposes that the manufacturers’ emission reduction level and the retailers’ low-carbon promotion level are linearly separable. In practice, the two types of effort are complementary. Therefore, the complementary effect is able to be considered in the future. Thus, future studies can extend this study by considering more complex decision conditions by the similar approaches; we believe that investigating the impact of low-carbon preference offers a fertile avenue on operation research and regulation policy in future.

## Figures and Tables

**Table 1:** Notations for the parameters and variables.

**Model Parameters**	**Underling Meaning of the Model Parameters**
*V*	Consumers’ valuation for the regular product
*t*	Consumers’ travelling cost
*τ*	Consumers’ preference for low-carbon products
*c_i_*	Cost of regular production
*e*	Carbon emission reduction rate
*k_i_*	Per unit fare to take low-carbon production
*ξ*	Promotional sensitivity coefficient
*θ_i_*	Retailers’ low-carbon promotional efforts
*U_i_*	Consumer utility
*Π_i_*	Profit of manufacturer/retailer
*D_i_*	Demand of products
**Decision Parameters**	**Underling Meaning of the Decision Parameters**
*p_i_*	Unit price
*w_i_*	Unit wholesale price

**Table 2:** Strategy selection game matrix of the two supply chains.

Scenarios	Supply Chain 1 (Regular Products)	Supply Chain 1 (Low-Carbon Products)
**Supply chain 2** (Regular products)	Scenario NN	Scenario LN
**Supply chain 2** (Low-carbon products)	Scenario NL	Scenario LL

**Table 3:** Equilibrium outcomes with subsidy under scenario NL.

Scenario NL	Supply Chain 1	Supply Chain 2
*w*	$\frac{2c_{1}+c_{2}+9t-\tau e-\xi \theta_{2}+\frac{1}{2}k_{2}e^{2}-\lambda e}{3}$	$\frac{c_{1}+2c_{2}+9t+\tau e+\xi \theta_{2}+k_{2}e^{2}-2\lambda e}{3}$
*p*	$\frac{5c_{1}+4c_{2}+36t-4\tau e-4\xi \theta_{2}+2k_{2}e^{2}-4\lambda e}{9}$	$\frac{4c_{1}+5c_{2}+36t+4\tau e+4\xi \theta_{1}+\frac{5}{2}k_{1}e^{2}-5\lambda e}{9}$
*D*	$\frac{c_{2}-c_{1}+9t-\tau e-\xi \theta_{2}+\frac{1}{2}k_{2}e^{2}-\lambda e}{18t}$	$\frac{c_{1}-c_{2}+9t+\tau e+\xi \theta_{2}-\frac{1}{2}k_{2}e^{2}+\lambda e}{18t}$
Π*_M_*	$\frac{{\left(c_{2}-c_{1}+9t-\tau e-\xi \theta_{2}+\frac{1}{2}k_{2}e^{2}-\lambda e\right)}^{2}}{54t}$	$\frac{{\left(c_{1}-c_{2}+9t+\tau e+\xi \theta_{2}-\frac{1}{2}k_{2}e^{2}+\lambda e\right)}^{2}}{54t}$
Π*_R_*	$\frac{{\left(c_{2}-c_{1}+9t-\tau e-\xi \theta_{2}+\frac{1}{2}k_{2}e^{2}-\lambda e\right)}^{2}}{162t}$	$\frac{{\left(c_{1}-c_{2}+9t+\tau e+\xi \theta_{2}-\frac{1}{2}k_{2}e^{2}+\lambda e\right)}^{2}-81tu_{2}\theta_{2}{}^{2}}{162t}$

**Table 4:** Equilibrium outcomes with subsidy under scenario LN.

Scenario LN	Supply Chain 1	Supply Chain 2
*w*	$\frac{2c_{1}+c_{2}+9t+\tau e+\xi \theta_{1}+k_{1}e^{2}-2\lambda e}{3}$	$\frac{c_{1}+2c_{2}+9t-\tau e-\xi \theta_{1}+\frac{1}{2}k_{1}e^{2}-\lambda e}{3}$
*p*	$\frac{5c_{1}+4c_{2}+36t+4\tau e+4\xi \theta_{1}+\frac{5}{2}k_{1}e^{2}-5\lambda e}{9}$	$\frac{4c_{1}+5c_{2}+36t-4\tau e-4\xi \theta_{1}+2k_{1}e^{2}-4\lambda e}{9}$
*D*	$\frac{c_{2}-c_{1}+9t+\tau e+\xi \theta_{1}-\frac{1}{2}k_{1}e^{2}+\lambda e}{18t}$	$\frac{c_{1}-c_{2}+9t-\tau e-\xi \theta_{1}+\frac{1}{2}k_{1}e^{2}-\lambda e}{18t}$
Π*_M_*	$\frac{{\left(c_{2}-c_{1}+9t+\tau e+\xi \theta_{1}-\frac{1}{2}k_{1}e^{2}+\lambda e\right)}^{2}}{54t}$	$\frac{{\left(c_{1}-c_{2}+9t-\tau e-\xi \theta_{1}+\frac{1}{2}k_{1}e^{2}-\lambda e\right)}^{2}}{54t}$
Π*_R_*	$\frac{{\left(c_{2}-c_{1}+9t+\tau e+\xi \theta_{1}-\frac{1}{2}k_{1}e^{2}+\lambda e\right)}^{2}-81tu_{1}\theta_{1}{}^{2}}{162t}$	$\frac{{\left(c_{1}-c_{2}+9t-\tau e-\xi \theta_{1}+\frac{1}{2}k_{1}e^{2}-\lambda e\right)}^{2}}{162t}$

**Table 5:** Equilibrium outcomes with subsidy under scenario LL.

Scenario LL	Supply Chain 1	Supply Chain 2
*w*	$\frac{2c_{1}+c_{2}+9t+\xi (\theta_{1}-\theta_{2})+k_{1}e^{2}+\frac{1}{2}k_{2}e^{2}}{3}-\lambda e$	$\frac{c_{1}+2c_{2}+9t-\xi (\theta_{1}-\theta_{2})+\frac{1}{2}k_{1}e^{2}+k_{2}e^{2}}{3}-\lambda e$
*p*	$\frac{5c_{1}+4c_{2}+36t+4\xi (\theta_{1}-\theta_{2})+\frac{5}{2}k_{1}e^{2}+2k_{2}e^{2}}{9}-\lambda e$	$\frac{4c_{1}+5c_{2}+36t-4\xi (\theta_{1}-\theta_{2})+2k_{1}e^{2}+\frac{5}{2}k_{2}e^{2}}{9}-\lambda e$
*D*	$\frac{c_{2}-c_{1}+9t+\xi (\theta_{1}-\theta_{2})-\frac{1}{2}k_{1}e^{2}+\frac{1}{2}k_{2}e^{2}}{18t}$	$\frac{c_{1}-c_{2}+9t-\xi (\theta_{1}-\theta_{2})+\frac{1}{2}k_{1}e^{2}-\frac{1}{2}k_{2}e^{2}}{18t}$
Π*_M_*	$\frac{{\left[c_{2}-c_{1}+9t+\xi (\theta_{1}-\theta_{2})-\frac{1}{2}k_{1}e^{2}+\frac{1}{2}k_{2}e^{2}\right]}^{2}}{54t}$	$\frac{{\left[c_{1}-c_{2}+9t-\xi (\theta_{1}-\theta_{2})+\frac{1}{2}k_{1}e^{2}-\frac{1}{2}k_{2}e^{2}\right]}^{2}}{54t}$
Π*_R_*	$\frac{{\left[c_{2}-c_{1}+9t+\xi (\theta_{1}-\theta_{2})-\frac{1}{2}k_{1}e^{2}+\frac{1}{2}k_{2}e^{2}\right]}^{2}-81tu_{1}\theta_{1}{}^{2}}{162t}$	$\frac{{\left(c_{1}-c_{2}+9t-\tau e-\xi \theta_{1}+\frac{1}{2}k_{1}e^{2}-\lambda e\right)}^{2}}{162t}$

## Appendix A

Equilibrium solutions of supply chains under different decision scenarios are calculated and presented in the following context below.

### Appendix A.1. Solutions under Scenario NN

By solving $U_{1}^{NN}=U_{2}^{NN}$, we can derive $x=\frac{t-p_{1}^{NN}+p_{2}^{NN}}{2t}$. The demand functions of the two retailers are $D_{1}^{NN}=x=\frac{t-p_{1}^{NN}+p_{2}^{NN}}{2t}$ and $D_{2}^{NN}=1-x=\frac{t+p_{1}^{NN}-p_{2}^{NN}}{2t}$.

The profits of the two manufacturers and two retailers can be modeled as follows based on the previously presented demand functions:

(A1) $$\Pi_{M1}^{NN}=(w_{1}^{NN}-c_{1})D_{1}^{NN}\;\mathrm{and}\;\Pi_{M2}^{NN}=(w_{2}^{NN}-c_{2})D_{2}^{NN},$$

(A2) $$\Pi_{R1}^{NN}=(p_{1}^{NN}-w_{1}^{NN})D_{1}^{NN}\;\mathrm{and}\;\Pi_{R2}^{NN}=(p_{2}^{NN}-w_{2}^{NN})D_{2}^{NN}.$$

By solving equations $\frac{\partial \Pi_{R1}^{NN}}{p_{1}^{NN}}=0$ and $\frac{\partial \Pi_{R2}^{NN}}{p_{2}^{NN}}=0$, we can obtain the following optimal functions: $p_{1}^{NN}=\frac{2w_{1}+w_{2}+3t}{3}$ and $p_{2}^{NN}=\frac{w_{1}+2w_{2}+3t}{3}$.

After substituting the function of $p_{1}^{NN}$ and $p_{2}^{NN}$ into $D_{1}^{NN}$ and $D_{2}^{NN}$ and solving equations $\frac{\partial \Pi_{M1}^{NN}}{w_{1}^{NN}}=0$ and $\frac{\partial \Pi_{M2}^{NN}}{w_{2}^{NN}}=0$, we can derive:

(A3) $$w_{1}^{NN*}=\frac{2c_{1}+c_{2}+9t}{3}\;\mathrm{and}\;w_{2}^{NN*}=\frac{c_{1}+2c_{2}+9t}{3}.$$

By substituting $w_{1}^{NN*}$ and $w_{2}^{NN*}$ into the equations about $p_{1}^{NN}$ and $p_{2}^{NN}$, we can derive

(A4) $$p_{1}^{NN*}=\frac{5c_{1}+4c_{2}+36t}{9}\;\mathrm{and}\;p_{2}^{NN*}=\frac{4c_{1}+5c_{2}+36t}{9}.$$

By substituting $p_{1}^{NN*}$ and $p_{2}^{NN*}$ into the equations on $D_{1}^{NN}$ and $D_{2}^{NN}$, we can obtain

(A5) $$D_{1}^{NN}=\frac{c_{2}-c_{1}+9t}{18t}\;\mathrm{and}\;D_{2}^{NN}=\frac{c_{1}-c_{2}+9t}{18t}.$$

By substituting the optimal solution into the equations about profits of the two manufacturers and two retailers, we can obtain

(A6) $$\Pi_{M1}^{NN}=\frac{{\left(c_{2}-c_{1}+9t\right)}^{2}}{54t}\;\mathrm{and}\;\Pi_{M2}^{NN}=\frac{{\left(c_{1}-c_{2}+9t\right)}^{2}}{54t},$$

(A7) $$\Pi_{R1}^{NN}=\frac{{\left(c_{2}-c_{1}+9t\right)}^{2}}{162t}\;\mathrm{and}\;\Pi_{R2}^{NN}=\frac{{\left(c_{1}-c_{2}+9t\right)}^{2}}{162t}.$$

Equilibrium solutions under scenario NN are summarized and presented in Table A1.

**Table A1:** Equilibrium outcomes under scenario NN.

Scenario NN	Supply Chain 1	Supply Chain 2
w	$\frac{2c_{1}+c_{2}+9t}{3}$	$\frac{c_{1}+2c_{2}+9t}{3}$
p	$\frac{5c_{1}+4c_{2}+36t}{9}$	$\frac{4c_{1}+5c_{2}+36t}{9}$
D	$\frac{c_{2}-c_{1}+9t}{18t}$	$\frac{c_{1}-c_{2}+9t}{18t}$
$\Pi_{M}$	$\frac{{\left(c_{2}-c_{1}+9t\right)}^{2}}{54t}$	$\frac{{\left(c_{1}-c_{2}+9t\right)}^{2}}{54t}$
$\Pi_{R}$	$\frac{{\left(c_{2}-c_{1}+9t\right)}^{2}}{162t}$	$\frac{{\left(c_{1}-c_{2}+9t\right)}^{2}}{162t}$

### Appendix A.2. Solutions under Scenario NL

By solving $U_{1}^{NL}=U_{2}^{NL}$, we can derive $x=\frac{t-p_{1}^{NL}+p_{2}^{NL}-\tau e-\xi \theta_{2}}{2t}$. The demand functions of the two retailers are as follows: $D_{1}^{NL}=x=\frac{t-p_{1}^{NL}+p_{2}^{NL}-\tau e-\xi \theta_{2}}{2t}$ and $D_{2}^{NL}=1-x=\frac{t+p_{1}^{NL}-p_{2}^{NL}+\tau e+\xi \theta_{2}}{2t}$.

The profits of the two manufacturers and two retailers can be modeled as follows based on the previously presented demand functions:

(A8) $$\Pi_{M1}^{NL}=(w_{1}^{NL}-c_{1})D_{1}^{NL}\;\mathrm{and}\;\Pi_{M2}^{NL}=(w_{2}^{NL}-c_{2}-\frac{1}{2}k_{2}e^{2})D_{2}^{NL},$$

(A9) $$\Pi_{R1}^{NL}=(p_{1}^{NL}-w_{1}^{NL})D_{1}^{NL}\;\mathrm{and}\;\Pi_{R2}^{NL}=(p_{2}^{NL}-w_{2}^{NL})D_{2}^{NL}-\frac{1}{2}u_{2}\theta_{2}{}^{2}.$$

By solving equations $\frac{\partial \Pi_{R1}^{NL}}{p_{1}^{NL}}=0$ and $\frac{\partial \Pi_{R2}^{NL}}{p_{2}^{NL}}=0$, we can obtain the following optimal functions:

(A10) $$p_{1}^{NL}=\frac{2w_{1}{}^{NL}+w_{2}{}^{NL}+3t-\tau e-\xi \theta_{2}}{3}\;\mathrm{and}\;p_{2}^{NL}=\frac{w_{1}{}^{NL}+2w_{2}{}^{NL}+3t+\tau e+\xi \theta_{2}}{3}.$$

After substituting the function of $p_{1}^{NL}$ and $p_{2}^{NL}$ into $D_{1}^{NL}$ and $D_{2}^{NL}$ and solving equations $\frac{\partial \Pi_{M1}^{NL}}{w_{1}^{NL}}=0$ and $\frac{\partial \Pi_{M2}^{NL}}{w_{2}^{NL}}=0$, we can derive

(A11) $$w_{1}^{NL*}=\frac{2c_{1}+c_{2}+9t-\tau e-\xi \theta_{2}+\frac{1}{2}k_{2}e^{2}}{3}\;\mathrm{and}\;w_{2}^{NL*}=\frac{c_{1}+2c_{2}+9t+\tau e+\xi \theta_{2}+k_{2}e^{2}}{3}\;$$

By substituting $w_{1}^{NL*}$ and $w_{2}^{NL*}$ into the equations about $p_{1}^{NL}$ and $p_{2}^{NL}$, we can derive

(A12) $$p_{1}^{NL*}=\frac{5c_{1}+4c_{2}+36t-4\tau e-4\xi \theta_{2}+2k_{2}e^{2}}{9}\;\mathrm{and}\;p_{2}^{NL*}=\frac{4c_{1}+5c_{2}+36t+4\tau e+4\xi \theta_{1}+\frac{5}{2}k_{1}e^{2}}{9}\;$$

By substituting $p_{1}^{NL*}$ and $p_{2}^{NL*}$ into the equations on $D_{1}^{NL}$ and $D_{2}^{NL}$, we can obtain

(A13) $$D_{1}^{NL}=\frac{c_{2}-c_{1}+9t-\tau e-\xi \theta_{2}+\frac{1}{2}k_{2}e^{2}}{18t}\;\mathrm{and}\;D_{2}^{NL}=\frac{c_{1}-c_{2}+9t+\tau e+\xi \theta_{2}-\frac{1}{2}k_{2}e^{2}}{18t}.$$

By substituting the optimal solution into the equations about profits of the two manufacturers and two retailers, we can obtain the following equations:

(A14) $$\Pi_{M1}^{NL}=\frac{{\left(c_{2}-c_{1}+9t-\tau e-\xi \theta_{2}+\frac{1}{2}k_{2}e^{2}\right)}^{2}}{54t}\;\mathrm{and}\;\Pi_{M2}^{NL}=\frac{{\left(c_{1}-c_{2}+9t+\tau e+\xi \theta_{2}-\frac{1}{2}k_{2}e^{2}\right)}^{2}}{54t},$$

(A15) $$\Pi_{R1}^{NL}=\frac{{\left(c_{2}-c_{1}+9t-\tau e-\xi \theta_{2}+\frac{1}{2}k_{2}e^{2}\right)}^{2}}{162t}\;\mathrm{and}\;\Pi_{R2}^{NL}=\frac{{\left(c_{1}-c_{2}+9t+\tau e+\xi \theta_{2}-\frac{1}{2}k_{2}e^{2}\right)}^{2}-81tu_{2}\theta_{2}{}^{2}}{162t}.$$

We summarize equilibrium solutions under scenario NL and presented in Table A2.

**Table A2:** Equilibrium outcomes under scenario NL.

Scenario NL	Supply Chain 1	Supply Chain 2
w	$\frac{2c_{1}+c_{2}+9t-\tau e-\xi \theta_{2}+\frac{1}{2}k_{2}e^{2}}{3}$	$\frac{c_{1}+2c_{2}+9t+\tau e+\xi \theta_{2}+k_{2}e^{2}}{3}$
p	$\frac{5c_{1}+4c_{2}+36t-4\tau e-4\xi \theta_{2}+2k_{2}e^{2}}{9}$	$\frac{4c_{1}+5c_{2}+36t+4\tau e+4\xi \theta_{1}+\frac{5}{2}k_{1}e^{2}}{9}$
D	$\frac{c_{2}-c_{1}+9t-\tau e-\xi \theta_{2}+\frac{1}{2}k_{2}e^{2}}{18t}$	$\frac{c_{1}-c_{2}+9t+\tau e+\xi \theta_{2}-\frac{1}{2}k_{2}e^{2}}{18t}$
$\Pi_{M}$	$\frac{{\left(c_{2}-c_{1}+9t-\tau e-\xi \theta_{2}+\frac{1}{2}k_{2}e^{2}\right)}^{2}}{54t}$	$\frac{{\left(c_{1}-c_{2}+9t+\tau e+\xi \theta_{2}-\frac{1}{2}k_{2}e^{2}\right)}^{2}}{54t}$
$\Pi_{R}$	$\frac{{\left(c_{2}-c_{1}+9t-\tau e-\xi \theta_{2}+\frac{1}{2}k_{2}e^{2}\right)}^{2}}{162t}$	$\frac{{\left(c_{1}-c_{2}+9t+\tau e+\xi \theta_{2}-\frac{1}{2}k_{2}e^{2}\right)}^{2}-81tu_{2}\theta_{2}{}^{2}}{162t}$

### Appendix A.3. Solutions under Scenario LN

By solving $U_{1}^{LN}=U_{2}^{LN}$, we can derive $x=\frac{t-p_{1}^{LN}+p_{2}^{LN}+\tau e+\xi \theta_{1}}{2t}$. The demand functions of the two retailers are as follows:

(A16) $$D_{1}^{LN}=x=\frac{t-p_{1}^{LN}+p_{2}^{LN}+\tau e+\xi \theta_{1}}{2t}\;\mathrm{and}\;D_{2}^{LN}=1-x=\frac{t+p_{1}^{LN}-p_{2}^{LN}-\tau e-\xi \theta_{1}}{2t}.$$

The profits of the two manufacturers and two retailers can be modeled as follows based on the previously presented demand functions:

(A17) $$\Pi_{M1}^{LN}=(w_{1}^{LN}-c_{1}-\frac{1}{2}k_{1}e^{2})D_{1}^{LN}\;\mathrm{and}\;\Pi_{M2}^{LN}=(w_{2}^{LN}-c_{2})D_{2}^{LN},$$

(A18) $$\Pi_{R1}^{LN}=(p_{1}^{LN}-w_{1}^{LN})D_{1}^{LN}-\frac{1}{2}u_{1}\theta_{1}{}^{2}\;\mathrm{and}\;\Pi_{R2}^{LN}=(p_{2}^{LN}-w_{2}^{LN})D_{2}^{LN}\;$$

By solving equations $\frac{\partial \Pi_{R1}^{LN}}{p_{1}^{LN}}=0$ and $\frac{\partial \Pi_{R2}^{LN}}{p_{2}^{LN}}=0$, we can obtain the optimal functions

(A19) $$p_{1}^{LN}=\frac{2w_{1}{}^{LN}+w_{2}{}^{LN}+3t+\tau e+\xi \theta_{1}}{3}\;\mathrm{and}\;p_{2}^{LN}=\frac{w_{1}{}^{LN}+2w_{2}{}^{LN}+3t-\tau e-\xi \theta_{1}}{3}.$$

After substituting the function of $p_{1}^{LN}$ and $p_{2}^{LN}$ into $D_{1}^{LN}$ and $D_{2}^{LN}$ and solving equations $\frac{\partial \Pi_{N1}^{LN}}{w_{1}^{LN}}=0$ and $\frac{\partial \Pi_{N2}^{LN}}{w_{2}^{LN}}=0$, we can derive

(A20) $$w_{1}^{LN*}=\frac{2c_{1}+c_{2}+9t+\tau e+\xi \theta_{1}+k_{1}e^{2}}{3}\;\mathrm{and}\;w_{2}^{LN*}=\frac{c_{1}+2c_{2}+9t-\tau e-\xi \theta_{1}+\frac{1}{2}k_{1}e^{2}}{3}\;$$

By substituting $w_{1}^{LN*}$ and $w_{2}^{LN*}$ into the equations about $p_{1}^{LN}$ and $p_{2}^{LN}$, we can derive

(A21) $$p_{1}^{LN*}=\frac{5c_{1}+4c_{2}+36t+4\tau e+4\xi \theta_{1}+\frac{5}{2}k_{1}e^{2}}{9}\;\mathrm{and}\;p_{2}^{LN*}=\frac{4c_{1}+5c_{2}+36t-4\tau e-4\xi \theta_{1}+2k_{1}e^{2}}{9}.$$

By substituting $p_{1}^{LN*}$ and $p_{2}^{LN*}$ into the equations on $D_{1}^{LN}$ and $D_{2}^{LN}$, we can obtain

(A22) $$D_{1}^{LN}=\frac{c_{2}-c_{1}+9t+\tau e+\xi \theta_{1}-\frac{1}{2}k_{1}e^{2}}{18t}\;\mathrm{and}\;D_{2}^{LN}=\frac{c_{1}-c_{2}+9t-\tau e-\xi \theta_{1}+\frac{1}{2}k_{1}e^{2}}{18t}.$$

By substituting the optimal solution into the equations about profits of the two manufacturers and two retailers, we can obtain the following equations:

(A23) $$\Pi_{M1}^{LN}=\frac{{\left(c_{2}-c_{1}+9t+\tau e+\xi \theta_{1}-\frac{1}{2}k_{1}e^{2}\right)}^{2}}{54t}\;\mathrm{and}\;\Pi_{M2}^{LN}=\frac{{\left(c_{1}-c_{2}+9t-\tau e-\xi \theta_{1}+\frac{1}{2}k_{1}e^{2}\right)}^{2}}{54t},$$

(A24) $$\Pi_{R1}^{LN}=\frac{{\left(c_{2}-c_{1}+9t+\tau e+\xi \theta_{1}-\frac{1}{2}k_{1}e^{2}\right)}^{2}-81tu_{1}\theta_{1}{}^{2}}{162t}\;\mathrm{and}\;\Pi_{R2}^{LN}=\frac{{\left(c_{1}-c_{2}+9t-\tau e-\xi \theta_{1}+\frac{1}{2}k_{1}e^{2}\right)}^{2}}{162t}\;$$

Equilibrium solutions under scenario LN are summarized and presented in Table A3.

**Table A3:** Equilibrium outcomes under scenario LN.

Scenario LN	Supply Chain 1	Supply Chain 2
w	$\frac{2c_{1}+c_{2}+9t+\tau e+\xi \theta_{1}+k_{1}e^{2}}{3}$	$\frac{c_{1}+2c_{2}+9t-\tau e-\xi \theta_{1}+\frac{1}{2}k_{1}e^{2}}{3}$
p	$\frac{5c_{1}+4c_{2}+36t+4\tau e+4\xi \theta_{1}+\frac{5}{2}k_{1}e^{2}}{9}$	$\frac{4c_{1}+5c_{2}+36t-4\tau e-4\xi \theta_{1}+2k_{1}e^{2}}{9}$
D	$\frac{c_{2}-c_{1}+9t+\tau e+\xi \theta_{1}-\frac{1}{2}k_{1}e^{2}}{18t}$	$\frac{c_{1}-c_{2}+9t-\tau e-\xi \theta_{1}+\frac{1}{2}k_{1}e^{2}}{18t}$
$\Pi_{M}$	$\frac{{\left(c_{2}-c_{1}+9t+\tau e+\xi \theta_{1}-\frac{1}{2}k_{1}e^{2}\right)}^{2}}{54t}$	$\frac{{\left(c_{1}-c_{2}+9t-\tau e-\xi \theta_{1}+\frac{1}{2}k_{1}e^{2}\right)}^{2}}{54t}$
$\Pi_{R}$	$\frac{{\left(c_{2}-c_{1}+9t+\tau e+\xi \theta_{1}-\frac{1}{2}k_{1}e^{2}\right)}^{2}-81tu_{1}\theta_{1}{}^{2}}{162t}$	$\frac{{\left(c_{1}-c_{2}+9t-\tau e-\xi \theta_{1}+\frac{1}{2}k_{1}e^{2}\right)}^{2}}{162t}$

### Appendix A.4. Solutions under Scenario LL

By solving $U_{1}^{LL}=U_{2}^{LL}$, we can derive $x=\frac{t-p_{1}^{LL}+p_{2}^{LL}+\xi (\theta_{1}-\theta_{2})}{2t}$. The demand functions of the two retailers are as follows:

(A25) $$D_{1}^{LL}=x=\frac{t-p_{1}^{LL}+p_{2}^{LL}+\xi (\theta_{1}-\theta_{2})}{2t}\;\mathrm{and}\;D_{2}^{LL}=1-x=\frac{t+p_{1}^{LL}-p_{2}^{LL}-\xi (\theta_{1}-\theta_{2})}{2t}.$$

The profits of the two manufacturers and two retailers can be modeled as follows based on the previously presented demand functions:

(A26) $$\Pi_{M1}^{LL}=(w_{1}^{LL}-c_{1}-\frac{1}{2}k_{1}e^{2})D_{1}^{LL}\;\mathrm{and}\;\Pi_{M2}^{LL}=(w_{2}^{LL}-c_{2}-\frac{1}{2}k_{2}e^{2})D_{2}^{LL},$$

(A27) $$\Pi_{R1}^{LL}=(p_{1}^{LL}-w_{1}^{LL})D_{1}^{LL}-\frac{1}{2}u_{1}\theta_{1}{}^{2}\;\mathrm{and}\;\Pi_{R2}^{LL}=(p_{2}^{LL}-w_{2}^{LL})D_{2}^{LL}-\frac{1}{2}u_{2}\theta_{2}{}^{2}\;$$

By solving equations $\frac{\partial \Pi_{R1}^{LL}}{p_{1}^{LL}}=0$ and $\frac{\partial \Pi_{R2}^{LL}}{p_{2}^{LL}}=0$, we can obtain the optimal functions $p_{1}^{LL}=\frac{2w_{1}^{LL}+w_{2}^{LL}+3t+\xi (\theta_{1}-\theta_{2})}{3}$ and $p_{2}^{LL}=\frac{w_{1}^{LL}+2w_{2}^{LL}+3t-\xi (\theta_{1}-\theta_{2})}{3}$.

After substituting the function of $p_{1}^{LL}$ and $p_{2}^{LL}$ into $D_{1}^{LL}$ and $D_{2}^{LL}$ and solving equations $\frac{\partial \Pi_{M1}^{LL}}{w_{1}^{LL}}=0$ and $\frac{\partial \Pi_{M2}^{LL}}{w_{2}^{LL}}=0$, we can derive

(A28) $$w_{1}^{LL*}=\frac{2c_{1}+c_{2}+9t+\xi (\theta_{1}-\theta_{2})+k_{1}e^{2}+\frac{1}{2}k_{2}e^{2}}{3}\;\mathrm{and}\;w_{2}^{LL*}=\frac{c_{1}+2c_{2}+9t-\xi (\theta_{1}-\theta_{2})+\frac{1}{2}k_{1}e^{2}+k_{2}e^{2}}{3}\;$$

By substituting $w_{1}^{LL*}$ and $w_{2}^{LL*}$ into the equations about $p_{1}^{LL}$ and $p_{2}^{LL}$, we can derive

(A29) $$p_{1}^{LL*}=\frac{5c_{1}+4c_{2}+36t+4\xi (\theta_{1}-\theta_{2})+\frac{5}{2}k_{1}e^{2}+2k_{2}e^{2}}{9},\;p_{2}^{LL*}=\frac{4c_{1}+5c_{2}+36t-4\xi (\theta_{1}-\theta_{2})+2k_{1}e^{2}+\frac{5}{2}k_{2}e^{2}}{9}\;$$

By substituting $p_{1}^{LL*}$ and $p_{2}^{LL*}$ into the equations on $D_{1}^{LL}$ and $D_{2}^{LL}$, we can obtain

(A30) $$D_{1}^{LL}=\frac{c_{2}-c_{1}+9t+\xi (\theta_{1}-\theta_{2})-\frac{1}{2}k_{1}e^{2}+\frac{1}{2}k_{2}e^{2}}{18t}\;\mathrm{and}\;D_{2}^{LL}=\frac{c_{1}-c_{2}+9t-\xi (\theta_{1}-\theta_{2})+\frac{1}{2}k_{1}e^{2}-\frac{1}{2}k_{2}e^{2}}{18t}.$$

By substituting the optimal solution into the equations about profits of the two manufacturers and two retailers, we can obtain the following equations:

(A31) $$\Pi_{M1}^{LL}=\frac{{\left[c_{2}-c_{1}+9t+\xi (\theta_{1}-\theta_{2})-\frac{1}{2}k_{1}e^{2}+\frac{1}{2}k_{2}e^{2}\right]}^{2}}{54t},\;\Pi_{R1}^{LL}=\frac{{\left[c_{2}-c_{1}+9t+\xi (\theta_{1}-\theta_{2})-\frac{1}{2}k_{1}e^{2}+\frac{1}{2}k_{2}e^{2}\right]}^{2}-81tu_{1}\theta_{1}{}^{2}}{162t}.$$

(A32) $$\Pi_{M2}^{LL}=\frac{{\left[c_{1}-c_{2}+9t-\xi (\theta_{1}-\theta_{2})+\frac{1}{2}k_{1}e^{2}-\frac{1}{2}k_{2}e^{2}\right]}^{2}}{54t},\;\Pi_{R2}^{LL}=\frac{{\left[c_{1}-c_{2}+9t-\xi (\theta_{1}-\theta_{2})+\frac{1}{2}k_{1}e^{2}-\frac{1}{2}k_{2}e^{2}\right]}^{2}-81tu_{2}\theta_{2}{}^{2}}{162t}\;$$

We summarize equilibrium solutions under scenario LL and presented in Table A4.

**Table A4:** Equilibrium outcomes under scenario LL.

Scenario LL	Supply Chain 1	Supply Chain 2
w	$\frac{2c_{1}+c_{2}+9t+\xi (\theta_{1}-\theta_{2})+k_{1}e^{2}+\frac{1}{2}k_{2}e^{2}}{3}$	$\frac{c_{1}+2c_{2}+9t-\xi (\theta_{1}-\theta_{2})+\frac{1}{2}k_{1}e^{2}+k_{2}e^{2}}{3}$
p	$\frac{5c_{1}+4c_{2}+36t+4\xi (\theta_{1}-\theta_{2})+\frac{5}{2}k_{1}e^{2}+2k_{2}e^{2}}{9}$	$\frac{4c_{1}+5c_{2}+36t-4\xi (\theta_{1}-\theta_{2})+2k_{1}e^{2}+\frac{5}{2}k_{2}e^{2}}{9}$
D	$\frac{c_{2}-c_{1}+9t+\xi (\theta_{1}-\theta_{2})-\frac{1}{2}k_{1}e^{2}+\frac{1}{2}k_{2}e^{2}}{18t}$	$\frac{c_{1}-c_{2}+9t-\xi (\theta_{1}-\theta_{2})+\frac{1}{2}k_{1}e^{2}-\frac{1}{2}k_{2}e^{2}}{18t}$
$\Pi_{M}$	$\frac{{\left[c_{2}-c_{1}+9t+\xi (\theta_{1}-\theta_{2})-\frac{1}{2}k_{1}e^{2}+\frac{1}{2}k_{2}e^{2}\right]}^{2}}{54t}$	$\frac{{\left[c_{2}-c_{1}+9t+\xi (\theta_{1}-\theta_{2})-\frac{1}{2}k_{1}e^{2}+\frac{1}{2}k_{2}e^{2}\right]}^{2}-81tu_{1}\theta_{1}{}^{2}}{162t}$
$\Pi_{R}$	$\frac{{\left[c_{1}-c_{2}+9t-\xi (\theta_{1}-\theta_{2})+\frac{1}{2}k_{1}e^{2}-\frac{1}{2}k_{2}e^{2}\right]}^{2}}{54t}$	$\frac{{\left[c_{1}-c_{2}+9t-\xi (\theta_{1}-\theta_{2})+\frac{1}{2}k_{1}e^{2}-\frac{1}{2}k_{2}e^{2}\right]}^{2}-81tu_{2}\theta_{2}{}^{2}}{162t}$

Next, we analyze the choice of manufacturers. Table A5 lists the equilibrium profit between manufacturers 1 and 2 under the four scenarios.

**Table A5:** Equilibrium profit of the two manufacturers under the four scenarios.

	NN	NL	LN	LL
M_1_	$\frac{M^{2}}{54t}$	$\frac{{\left(M-\tau e-\xi \theta_{2}+\frac{1}{2}k_{2}e^{2}\right)}^{2}}{54t}$	$\frac{{\left(M+\tau e+\xi \theta_{1}-\frac{1}{2}k_{1}e^{2}\right)}^{2}}{54t}$	$\frac{{\left[M+\xi (\theta_{1}-\theta_{2})-\frac{1}{2}k_{1}e^{2}+\frac{1}{2}k_{2}e^{2}\right]}^{2}}{54t}$
M_2_	$\frac{N^{2}}{54t}$	$\frac{{\left(N+\tau e+\xi \theta_{2}-\frac{1}{2}k_{2}e^{2}\right)}^{2}}{54t}$	$\frac{{\left(N-\tau e-\xi \theta_{1}+\frac{1}{2}k_{1}e^{2}\right)}^{2}}{54t}$	$\frac{{\left[N-\xi (\theta_{1}-\theta_{2})+\frac{1}{2}k_{1}e^{2}-\frac{1}{2}k_{2}e^{2}\right]}^{2}}{54t}$

Where *M* = *C*_2_ − *C*_1_ + 9*t*, *N* = *C*_1_ − *C*_2_ + 9*t*.

## Appendix B

Appendix B contains the proofs of the propositions and lemmas stated in the text.

**Proof** **of** **Proposition** **1.** Due to e>0, $k_{1}<k_{2}$, and $\theta_{1}>\theta_{2}$, we can derive $\frac{1}{2}k_{1}e-\frac{\xi \theta_{1}}{e}<\frac{1}{2}k_{2}e-\frac{\xi \theta_{2}}{e}$; thus, A<B.

Manufacturer 1’s profit gap between scenario NN and scenario LN is

(A33) $$\Pi_{M1}^{NN}-\Pi_{M1}^{LN}=\frac{\left[M+(M+\tau e+\xi \theta_{1}-\frac{1}{2}k_{1}e^{2})\right]\left[M-(M+\tau e+\xi \theta_{1}-\frac{1}{2}k_{1}e^{2})\right]}{54t}.$$

Due to $D_{1}^{NN}=\frac{M}{18t}>0$, $D_{1}^{LN}=\frac{M+\tau e+\xi \theta_{1}-\frac{1}{2}k_{1}e^{2}}{18t}>0$, t>0, we can derive M>0,

(A34) $$M+\tau e+\xi \theta_{1}-\frac{1}{2}k_{1}e^{2}>0;\;\mathrm{then},\;(M+\tau e+\xi \theta_{1}-\frac{1}{2}k_{1}e^{2})+M>0.$$

(A35) $$M-(M+\tau e+\xi \theta_{1}-\frac{1}{2}k_{1}e^{2})=\frac{1}{2}k_{1}e^{2}-\tau e-\xi \theta_{1}\;$$

When $\Pi_{M1}^{NN}>\Pi_{M1}^{LN}$, $\frac{1}{2}k_{1}e^{2}-\tau e-\xi \theta_{1}>0$. Thus, $\tau <\frac{1}{2}k_{1}e-\frac{\xi \theta_{1}}{e}$.

In the same manner, we can determined that when $\Pi_{M1}^{NL}>\Pi_{M1}^{LL}$, $\tau <\frac{1}{2}k_{1}e-\frac{\xi \theta_{1}}{e}$.

Thus, if $\tau <\frac{1}{2}k_{1}e-\frac{\xi \theta_{1}}{e}$, regardless of what manufacturer 2 chooses to produce, then manufacturer 1 will choose to produce regular products.

In the same manner, we can determine that when $\Pi_{M2}^{NN}>\Pi_{M2}^{NL}$, $\tau <\frac{1}{2}k_{2}e-\frac{\xi \theta_{2}}{e}$; when $\Pi_{M2}^{LN}>\Pi_{M2}^{LL}$, $\tau <\frac{1}{2}k_{2}e-\frac{\xi \theta_{2}}{e}$. This finding means that if $\tau <\frac{1}{2}k_{2}e-\frac{\xi \theta_{2}}{e}$, regardless of what manufacturer 1 chooses to produce, then manufacturer 2 will choose to produce regular products.

Due to $\frac{1}{2}k_{1}e-\frac{\xi \theta_{1}}{e}<\frac{1}{2}k_{2}e-\frac{\xi \theta_{2}}{e}$, when $\tau <\frac{1}{2}k_{1}e-\frac{\xi \theta_{1}}{e}$, the equilibrium scenario is NN.

Similarly, when $\frac{1}{2}k_{1}e-\frac{\xi \theta_{1}}{e}\leq \tau <\frac{1}{2}k_{2}e-\frac{\xi \theta_{2}}{e}$, the equilibrium scenario is LN; when $\tau \geq \frac{1}{2}k_{2}e-\frac{\xi \theta_{2}}{e}$, the equilibrium scenario is LL.

However, when $\frac{1}{2}k_{2}e-\frac{\xi \theta_{2}}{e}<\tau <\frac{1}{2}k_{1}e-\frac{\xi \theta_{1}}{e}$, the equilibrium scenario should be NL, although because of $\frac{1}{2}k_{1}e-\frac{\xi \theta_{1}}{e}<\frac{1}{2}k_{2}e-\frac{\xi \theta_{2}}{e}$, this equilibrium will not exist.

**Proof** **of** **Lemma** **1.** $\Delta D^{NN}=D_{1}^{NN}-D_{2}^{NN}=\frac{c_{2}-c_{1}+9t}{18t}-\frac{c_{1}-c_{2}+9t}{18t}=\frac{c_{2}-c_{1}}{9t}$.

(A36) $$\Delta D^{LN}=D_{1}^{LN}-D_{2}^{LN}=\frac{c_{2}-c_{1}+9t+\tau e+\xi \theta_{1}-\frac{1}{2}k_{1}e^{2}}{18t}-\frac{c_{1}-c_{2}+9t-\tau e-\xi \theta_{1}+\frac{1}{2}k_{1}e^{2}}{18t}=\frac{c_{2}-c_{1}+\tau e+\xi \theta_{1}-\frac{1}{2}k_{1}e^{2}}{9t}\;$$

(A37) $$\Delta D^{LL}=D_{1}^{LL}-D_{2}^{LL}=\frac{c_{2}-c_{1}+9t+\xi (\theta_{1}-\theta_{2})-\frac{1}{2}k_{1}e^{2}+\frac{1}{2}k_{2}e^{2}}{18t}-\frac{c_{1}-c_{2}+9t-\xi (\theta_{1}-\theta_{2})+\frac{1}{2}k_{1}e^{2}-\frac{1}{2}k_{2}e^{2}}{18t}=\frac{c_{2}-c_{1}+\xi (\theta_{1}-\theta_{2})-\frac{1}{2}k_{1}e^{2}+\frac{1}{2}k_{2}e^{2}}{9t}\;$$

First, the difference of the demand gap in scenarios LL and NN is determined as follows: $\Delta D^{LL}-\Delta D^{NN}=\frac{\xi (\theta_{1}-\theta_{2})+\frac{1}{2}(k_{2}-k_{1})e^{2}}{9t}$.

Given that $\theta_{1}>\theta_{2}$, $k_{2}>k_{1}$, ξ>0, t>0, $e^{2}>0$, $\frac{\xi (\theta_{1}-\theta_{2})+\frac{1}{2}(k_{2}-k_{1})e^{2}}{9t}>0$. Thus, $\Delta D^{LL}>\Delta D^{NN}$.

In the same manner, we can derive $\Delta D^{LL}-\Delta D^{LN}=\frac{\frac{1}{2}k_{2}e^{2}-\xi \theta_{2}-\tau e}{9t}$. When $\Delta D^{LL}\leq \Delta D^{LN}$ $\tau \geq \frac{1}{2}k_{2}e-\frac{\xi \theta_{2}}{e}$. In summary, when $\tau \geq \frac{1}{2}k_{2}e-\frac{\xi \theta_{2}}{e}$, $\Delta D^{NN}\leq \Delta D^{LL}\leq \Delta D^{LN}$.

Similarly, when $\frac{1}{2}k_{1}e-\frac{\xi \theta_{1}}{e}\leq \tau <\frac{1}{2}k_{2}e-\frac{\xi \theta_{2}}{e}$, we can derive $\Delta D^{NN}\leq \Delta D^{LN}<\Delta D^{LL}$; when $\tau <\frac{1}{2}k_{1}e-\frac{\xi \theta_{1}}{e}$, we can derive $\Delta D^{LN}<\Delta D^{NN}<\Delta D^{LL}$.

**Proof** **of** **Proposition** **2.** Manufacturer 1’s price gap between scenario LN and scenario NN is $p_{1}^{LN*}-p_{1}^{NN*}=\frac{4\tau e+4\xi \theta_{1}+\frac{5}{2}k_{1}e^{2}}{9}$. Given that τ>0, e>0, $\theta_{1}>0$, ξ>0, $k_{1}>0$, $e^{2}>0$, we can derive $\frac{4\tau e+4\xi \theta_{1}+\frac{5}{2}k_{1}e^{2}}{9}>0$. Thus, $p_{1}^{LN*}>p_{1}^{NN*}$ is constantly true.

Manufacturer 1’s price gap between scenario LL and scenario NN is $p_{1}^{LL*}-p_{1}^{NN*}=\frac{4\xi (\theta_{1}-\theta_{2})+\frac{5}{2}k_{1}e^{2}+2k_{2}e^{2}}{9}$. Given that ξ>0, $\theta_{1}>\theta_{2}$, $k_{1}>0$, $k_{2}>0$, $e^{2}>0$, we can derive $\frac{4\xi (\theta_{1}-\theta_{2})+\frac{5}{2}k_{1}e^{2}+2k_{2}e^{2}}{9}>0$. Thus, $p_{1}^{LL*}>p_{1}^{NN*}$ is constantly true.

Manufacturer 1’s price gap between scenario LL and scenario LN is $p_{1}^{LL*}-p_{1}^{LN*}=\frac{-4\xi \theta_{2}-4\tau e+2k_{2}e^{2}}{9}$. If $p_{1}^{LL*}>p_{1}^{LN*}$, $\frac{-4\xi \theta_{2}-4\tau e+2k_{2}e^{2}}{9}>0$, then we can derive $\tau <\frac{1}{2}k_{2}e-\frac{\xi \theta_{2}}{e}$; if $p_{1}^{LL*}<p_{1}^{LN*}$, then we can derive $\tau >\frac{1}{2}k_{2}e-\frac{\xi \theta_{2}}{e}$.

In summary, if τ<B, then $p_{1}^{NN*}<p_{1}^{LN*}<p_{1}^{LL*}$; if τ≥B, then $p_{1}^{NN*}<p_{1}^{LL*}<p_{1}^{LN*}$.

**Proof** **of** **Lemma** **2.** Manufacturer 1’s demand gap between scenario LL and scenario NN is $D_{1}^{LL}-D_{1}^{NN}=\frac{\xi (\theta_{1}-\theta_{2})+\frac{1}{2}(k_{2}-k_{1})e^{2}}{18t}$. Given that ξ>0, t>0, $\theta_{1}>\theta_{2}$ and $k_{2}>k_{1}$, thus $D_{1}^{LL}>D_{1}^{NN}$ is constantly true.

Manufacturer 1’s demand gap between scenario LL and scenario LN is:

$D_{1}^{LL}-D_{1}^{LN}=\frac{-\xi \theta_{2}-\tau e+\frac{1}{2}k_{2}e^{2}}{18t}$. If $D_{1}^{LL}<D_{1}^{LN}$, then $\frac{\frac{1}{2}k_{2}e^{2}-\xi \theta_{2}-\tau e}{18t}<0$, and we can derive $\tau >\frac{1}{2}k_{2}e-\frac{\xi \theta_{2}}{e}$.

In summary, when $\tau >\frac{1}{2}k_{2}e-\frac{\xi \theta_{2}}{e}$, $D_{1}^{NN}<D_{1}^{LL}<D_{1}^{LN}$.

Similarly, when $\frac{1}{2}k_{1}e-\frac{\xi \theta_{1}}{e}\leq \tau <\frac{1}{2}k_{2}e-\frac{\xi \theta_{2}}{e}$, we can derive $D_{1}^{NN}<D_{1}^{LN}<D_{1}^{LL}$; when $\tau <\frac{1}{2}k_{1}e-\frac{\xi \theta_{1}}{e}$, we can derive $D_{1}^{LN}<D_{1}^{NN}<D_{1}^{LL}$.

**Proof** **of** **Lemma** **3.** $\frac{\partial \Pi_{R1}^{LN}}{\partial \xi }=\frac{\theta_{1}(c_{2}-c_{1}+9t+\tau e+\xi \theta_{1}-\frac{1}{2}k_{1}e^{2})}{81t}$, given that $D_{1}^{LN*}=\frac{c_{2}-c_{1}+9t+\tau e+\xi \theta_{1}-\frac{1}{2}k_{1}e^{2}}{18t}>0$, $\theta_{1}>0$, t>0; then, $\frac{\partial \Pi_{R1}^{LN}}{\partial \xi }>0$.

Similarly, we can prove $\frac{\partial \Pi_{R2}^{LN*}}{\partial \xi }=\frac{-\theta_{1}(c_{1}-c_{2}+9t-\tau e-\xi \theta_{1}+\frac{1}{2}k_{1}e^{2})}{81t}<0$,

(A38) $$\frac{\Pi_{R1}^{LL*}}{\partial \xi }=\frac{(\theta_{1}-\theta_{2})\left[c_{2}-c_{1}+9t+\xi (\theta_{1}-\theta_{2})-\frac{1}{2}k_{1}e^{2}+\frac{1}{2}k_{2}e^{2}\right]}{81t}>0,$$

(A39) $$\frac{\Pi_{R2}^{LL*}}{\partial \xi }=\frac{(\theta_{2}-\theta_{1})\left[c_{1}-c_{2}+9t-\xi (\theta_{1}-\theta_{2})+\frac{1}{2}k_{1}e^{2}-\frac{1}{2}k_{2}e^{2}\right]}{81t}<0\;$$

**Proof** **of** **Lemma** **4.** $\frac{\partial p_{1}^{LL*}}{\partial e}=\frac{\left(5k_{1}+4k_{2}\right)e}{9}$, given that $k_{1}>0$, $k_{2}>0$, e>0, we can derive $\frac{\left(5k_{1}+4k_{2}\right)e}{9}>0$. Thus, $\frac{\partial p_{1}^{LL*}}{\partial e}>0$. In the same manner, we can derive $\frac{\partial p_{2}^{LL*}}{\partial e}=\frac{\left(4k_{1}+5k_{2}\right)e}{9}>0$ and $\frac{\partial p_{1}^{LN*}}{\partial e}=\frac{5k_{1}e+4\tau }{9}>0$.
